# Prevalence of Leishmaniasis among Blood Donors: A Systematic Review and Meta-Analysis

**DOI:** 10.3390/diseases12070160

**Published:** 2024-07-17

**Authors:** Maria Kantzanou, Evangelos Kostares, Georgia Kostare, Evangelia Papagiannopoulou, Michael Kostares, Athanasios Tsakris

**Affiliations:** 1 Department of Microbiology, Medical School, National and Kapodistrian University of Athens, 115 27 Athens, Greece; 2 Department of Anatomy, Medical School, National and Kapodistrian University of Athens, 115 27 Athens, Greece

**Keywords:** leishmaniasis, blood donors, prevalence, PCR, serological analysis, meta-analysis

## Abstract

Our study seeks to provide a comprehensive assessment of leishmaniasis prevalence among blood donors, employing rigorous methodologies to inform public health initiatives and transfusion safety measures. A thorough literature search was conducted using electronic databases (Medline, Scopus, Web of Science, and Google Scholar) to identify the relevant studies reporting the prevalence of leishmaniasis among blood donors, gathering a wide range of studies encompassing different geographic locations and time periods. The pooled prevalence with a 95% confidence interval (CI) was estimated, and quality assessment, outlier analysis, and influential analysis were performed to ensure the robustness and validity of the findings. Our search and subsequent analyses led to the inclusion of thirty-five studies in our review. Using molecular diagnostic methods, the prevalence was estimated at 2.3% (95% CI 1–3.9%), while serological diagnostic methods indicated a higher prevalence rate of 4.5% (95% CI 2.8–6.7%). Notably, we observed significant heterogeneity among the included studies for each analysis. The observed heterogeneity highlights the need for future research to delve into the factors influencing leishmaniasis prevalence, with prospective and retrospective studies addressing the limitations identified in this review.

## 1. Introduction

Leishmaniasis is a disease triggered by parasitic protozoa belonging to the genus Leishmania (family Trypanosomatidae). Typically, the transmission of leishmaniasis occurs when an infected female sand fly bites (approximately 30 species of phlebotomine sand flies) mammals, rodents, marsupials, edentates, monkeys, and both wild and domestic canines, serving as reservoirs for the disease. Specific sand fly species are associated with the transmission of particular species of the parasite. Humans residing in endemic areas can also become incidentally infected [1,2]. Although it is relatively uncommon, leishmaniasis can be spread through different routes of transmission, such as intravenous drug administration, blood transfusion, organ transplantation, congenital infection, and laboratory mishaps [2]. Leishmaniasis has had a significant historical impact, spreading extensively across various continents with tropical climates, encompassing Europe, Africa, Asia, and America. In humans, these parasitic organisms undergo intracellular replication and commonly result in three distinct syndromes: cutaneous leishmaniasis (CL), mucocutaneous leishmaniasis (MCL), and visceral leishmaniasis (VL) [1,3].

Leishmaniasis impacts a considerable number of people, with an estimated 12 million individuals affected globally. Each year, there are approximately 0.2–0.4 million new cases of VL and 0.7–1.2 million new cases of CL reported. Additionally, asymptomatic leishmaniasis is prevalent in around 11.2% (95%CI 8.6–14.4%) of the general population [4], while the prevalence of leishmaniasis among individuals living with HIV is estimated to be 6% (95%CI, 4–11%) [5].

Leishmaniasis initially manifests as erythema at the site of the insect bite, serving as a crucial indication of infection. The ensuing inflammatory response caused by the parasites can result in the formation of ulcers or dissemination to vital organs like the spleen and liver. Therefore, the early detection of leishmaniasis holds tremendous importance in preventing the development of severe clinical symptoms and reducing the mortality rates. Conventional diagnosis involves the microscopic examination of tissue samples or parasite culturing, but these methods have limitations. Various others diagnostic methods exist, including serological techniques, such as the enzyme-linked immunosorbent assay (ELISA), Western blotting (WB), the indirect fluorescent antibody test (IFAT), the direct agglutination test (DAT), and the indirect hemagglutination test (IHA), and molecular diagnostic approaches, such as conventional PCR, nested PCR, and real-time PCR. The choice of diagnostic tests for leishmaniasis among blood donors carries significant implications for disease management, public health policy, and resource allocation. The molecular diagnostic methods, such as PCR, offer high sensitivity and specificity, enabling the early and precise detection of leishmanial DNA. However, they are more costly and require specialized equipment and trained personnel. Serological tests, while simpler and cheaper, may yield false positive results, leading to the overestimation of disease prevalence [6,7,8,9].

Blood transfusion is a critical medical intervention employed in various emergency and elective procedures, as well as for patients with blood disorders. However, the inadvertent transmission of infectious agents, including parasites, remains a significant risk. The scientific literature reveals significant variability in the prevalence of leishmaniasis among blood donors. To address this knowledge gap, our systematic review and meta-analysis aim to provide a comprehensive assessment of the prevalence of leishmaniasis in this specific population. By synthesizing data from multiple studies, our research aims to uncover the true extent of this hidden infection within the donor population, offering valuable insights to the scientific community.

## 2. Materials and Methods

### 2.1. Search Strategy

**Figure 1:**
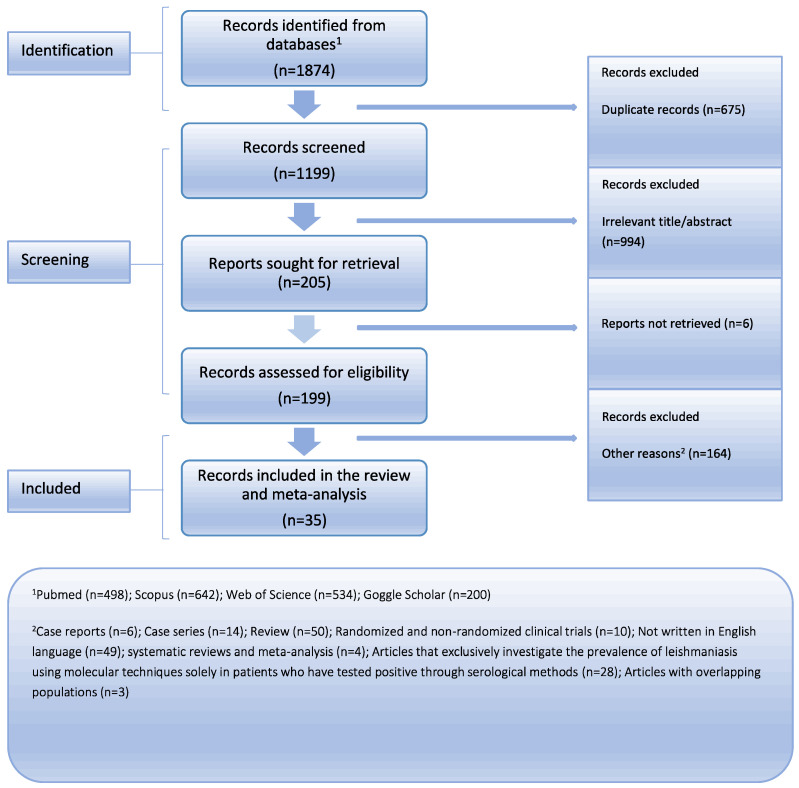
Flow chart depicting the systematic search results from the relevant studies’ identification and selection.

Medline (PubMed search engine), Scopus, Web of Science, and Google Scholar were comprehensively searched following the Preferred Reporting Items for Systematic Reviews and Meta-Analysis (PRISMA) guidelines to ensure a rigorous approach (Figure 1) [10]. The PRISMA checklist, available in the Supplementary Materials (Supplementary Table S1), was utilized to facilitate the systematic review process. We collected articles that were published up until 27 February 2024. Two reviewers independently conducted the literature search, employing a combination of the following keywords: “leishmaniasis”, “leishmania”, “blood donors”, “blood transfusion”, “blood bank”, “blood donate”, “prevalence”, “incidence”, and “rate”. The Supplementary Materials (Supplementary Table S2) provide the whole search method for every database. To find any further papers that could have been missed, a detailed review of the reference lists from the studies that were found was conducted in addition to the main search. Zotero reference management software (version 6.0.18) was used to carefully arrange and preserve the gathered research [11]. With great care, we eliminated any duplicate references from our dataset to guarantee its authenticity. Two separate detectives went through the remaining articles one by one after the first search. There were two separate steps in the study selection process. First, we carefully went through the article titles and abstracts, removing those that did not fit our preset inclusion criteria. In the subsequent phase, we acquired the whole manuscripts of the remaining papers and carried out exhaustive assessment. The team members reached a consensus to settle any disputes that arose throughout the research selection process, guaranteeing a uniform and cohesive decision-making procedure. Our objective in using this methodical technique was to obtain a thorough and trustworthy set of data for our analysis.

### 2.2. Criteria for Study Selection and Data Extraction

After conducting a thorough and extensive search across various databases, we carefully established our eligibility criteria based on the PECOS framework. This was conducted to guarantee clarity and precision in our systematic review and meta-analysis, which centers on the prevalence of Leishmaniasis among blood donors. Our review includes:

Population (P): Blood donors. This study focuses on assessing the prevalence of leishmaniasis among individuals who donate blood, aiming to gather data from a diverse group of participants across different geographic locations and time periods.

Exposure (E): The exposure under investigation is the presence of leishmaniasis in blood donors.

Comparison (C): Given that our objective was to quantify the prevalence of leishmaniasis among blood donors, a direct comparison component does not apply to our study’s framework.

Outcomes (O): The primary outcome of this study is the prevalence rate of leishmaniasis among blood donors, measured using molecular and serological diagnostic methods. The secondary outcomes include the identification of major risk factors associated with increased prevalence rates and the assessment of heterogeneity among the included studies.

Study Types (S): Our inclusion criteria encompassed solely observational studies, including cohort, case-control, and cross-sectional studies.

Inclusion Criteria: Articles that examined specifically the prevalence rates of Leishmaniasis among blood donors were included with no restriction on the publication date. Also, it is important to note that when considering the meta-analysis of the molecular method of diagnosis, we included studies that have implemented molecular diagnostic methods in the entire study population, rather than solely focusing on the individuals identified as positive through serological methods. This approach enables a more homogeneous population since a negative result from a serological method does not necessarily indicate a negative result from the molecular method.

Exclusion Criteria: We opted to omit certain categories of articles from consideration. These exclusions comprised case reports, case series involving fewer than five participants, review articles, randomized and non-randomized clinical trials [12], systematic reviews, meta-analyses, animal studies, books, expert opinions, conference abstracts, studies not written in English, articles that exclusively investigate the prevalence of leishmaniasis using molecular techniques solely in patients who have tested positive using serological methods, and studies lacking full-text accessibility. In situations where articles had overlapping populations, preference was given to the most recent or comprehensive publication for inclusion.

Data Extraction: For each included study, we gathered the following information: the primary author’s name, publication year, study design, continent of origin, country, study duration, total blood units, proportion of males, mean age, patients with leishmaniasis, and diagnostic procedure performed.

### 2.3. Quality Assessment

Two researchers independently conducted detailed evaluation of each study using the Quality Assessment Tools developed through a collaboration between the Universities of Newcastle, Australia, and Ottawa, Canada. They employed the Newcastle–Ottawa Scale (NOS) and its adapted version for cohort and cross-sectional studies. The goal was to identify potential methodological or survey implementation issues that could affect internal validity. The assessment used a ‘star system’ to evaluate studies on three main aspects: the selection of study groups, the comparability of the groups, and the determination of exposure or outcome of interest, depending on the study type. Studies scoring between 7 and 9 were considered to have low risk of bias (high quality), those scoring between 4 and 6 were deemed moderate quality, and scores from 0 to 3 indicated a high risk of bias (low quality) [13].

### 2.4. Statistical Analysis

RStudio software, specifically version 2022.12.0 + 353 (RStudio Team, 2022), was employed for conducting statistical analysis [14,15]. Meta-analysis was executed using the metafor software package [16]. The estimation of the pooled prevalence along with its corresponding 95% confidence interval (CI) was performed using the DerSimonian and Laird random effects model, which incorporated the Freeman–Tukey double arcsine transformation [17]. The presence of heterogeneity among the included studies was visually assessed by inspecting the forest plot and evaluated using both the associated *p*-value and Cochran’s Q statistic. Additionally, the I^2^ statistic was calculated to quantify the degree of heterogeneity. The extent of true heterogeneity in effect sizes was quantified with the Higgins I^2^ statistic and its associated 95% CI. According to the I^2^ values, 0–40%, 30–60%, 50–90%, and 75–100% represented not significant, moderate, significant, and substantial heterogeneity, respectively. To determine if any potentially outlying effect sizes were also influential, we performed screening for externally studentized residuals with z-values exceeding two in absolute value, as well as leave-one-out diagnostics [18]. Due to insufficient data, defined as having fewer than ten studies, for variables such as the mean age, these variables were not included in analysis [19]. Unless otherwise specified, statistical significance was determined at a *p*-value of 0.05 (two-tailed). Publication bias was assessed qualitatively due to the non-comparative nature of the data and the lack of a clear definition or consensus on what constitutes a positive result in meta-analyses of proportions. This qualitative assessment was necessitated by the inherent complexities and subjective interpretations associated with such data. Tests such as Egger’s test [20], Begg’s test [21], and funnel plots were developed to evaluate publication bias. These tests are based on the assumption that studies with significant findings are more likely to be published than those with non-significant results, leading to asymmetry in the distribution of the study outcomes [22].

## 3. Results

### 3.1. Results and Characteristics of the Included Studies

In total, thirty-five studies were finally included in this analysis. All the articles were published from 1991 to 2023 (conducted from 1996 to 2020). One of the studies was a cohort study, while the others employed a cross-sectional design. Geographically, the majority of these investigations were conducted in Europe (Spain, Italy, Greece, Monaco, and Portugal), Asia (Pakistan, Iran, Nepal, Bangladesh, and Turkey), South America (Brazil), Africa (Ethiopia and Sudan), and Oceania (Australia). With regard to the studies conducted to explore the prevalence of leishmaniasis through the utilization of molecular diagnostic techniques, such as real-time PCR, nested PCR, and conventional PCR, a total of fourteen studies involving 10,063 blood units were examined. This analysis indicated that males accounted for an average of 70.6% of the participants. In addition, thirty studies encompassing 24,359 blood units were included to investigate the prevalence of leishmaniasis using serological diagnostic procedures, namely WB, IHA, IFAT, ELISA, and DAT. Notably, collective analysis indicated that males accounted for an average of 74.2% of the participants, while the mean age varied from 27.7 years to 41 years, with a median age of 35.7 years. According to the quality assessment, six studies were evaluated as high-quality, while the rest were considered to be of moderate quality. The descriptive characteristics of them are reported in Table 1.

### 3.2. Prevalence of Leishmaniasis among Blood Donors

**Figure 2:**
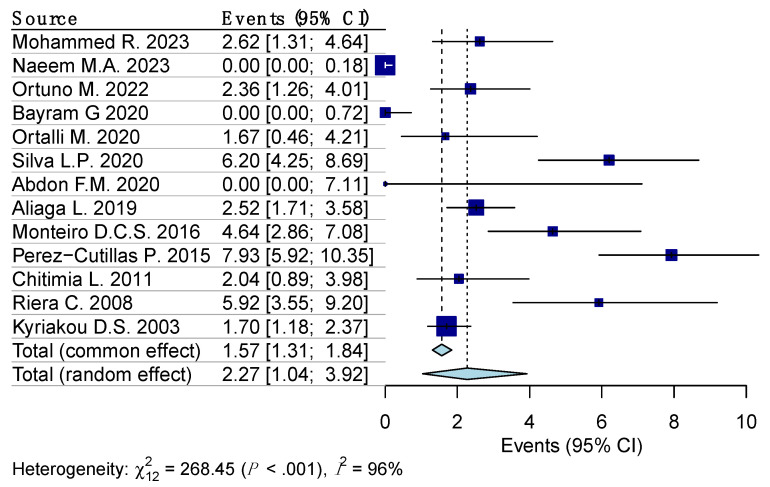
Forest plot evaluating the calculated prevalence of leishmaniasis among blood donors using random effects model (molecular diagnostic procedures).

Random effects model analysis revealed the prevalence of Leishmaniasis among the blood donors determined using molecular diagnostic methods, at 3.7% (95% CI 0.9–8.1%), accompanied by substantial heterogeneity between the studies (I^2^ = 99%, 95% CI 97.7–99.5%, *p* < 0.001). The influencing diagnostics and a forest plot illustrating the results of leave-one-out analysis are presented in the Supplementary Materials (Supplementary Figures S1 and S2). As per them, the study conducted by Asfaram S., et al. [30] was identified as influential. After the exclusion of the aforementioned study, the estimated prevalence was calculated at 2.3% (95% CI 1–3.9%), with a remaining substantial between-studies heterogeneity of I^2^ = 96% (95% CI 89.4–98.1%, *p* < 0.001) (Figure 2).

**Figure 3:**
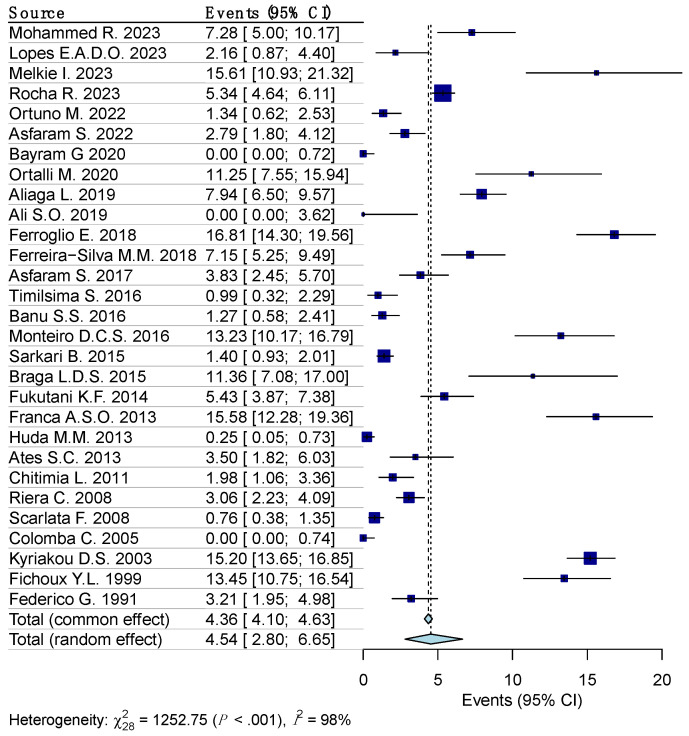
Forest plot evaluating the calculated prevalence of leishmaniasis among blood donors using random effects model (serological diagnostic procedures).

Random effects model analysis revealed the prevalence of leishmania reactivity among the blood donors determined using serological diagnostic methods, at 5.1% (95% CI 3.1–7.6%), accompanied by substantial heterogeneity between the studies (I^2^ = 98%, 95% CI 97.6–99.2%, *p* < 0.001). The influencing diagnostics and a forest plot illustrating the results of leave-one-out analysis are presented in the Supplementary Materials (Supplementary Figure S3 and S4). As per them, the study conducted by Panahi E., et al. [25] was identified as influential. After the exclusion of the aforementioned study, the estimated prevalence was calculated at 4.5% (95% CI 2.8–6.7%), with a remaining substantial between-studies heterogeneity of I^2^ = 98% (95% CI 96.8–93.5%, *p* < 0.001) (Figure 3).

## 4. Discussion

Our findings reveal the notable prevalence of leishmaniasis among the blood donors, as determined using both molecular and serological diagnostic methods. The prevalence estimates obtained were 2.3% (95% CI 1–3.9%) and 4.5% (95% CI 2.8–6.7%), respectively. However, it is imperative to acknowledge the presence of substantial heterogeneity between the studies. The substantial heterogeneity observed between the included studies may be attributed to several factors. First, it is worth emphasizing that the current studies are observational and were conducted in different locations, times, and conditions. Therefore, the variation in the geographical region and population under investigation can contribute to differences in the prevalence of leishmaniasis. This disease is endemic in various regions worldwide, with variations in transmission intensity and prevalence rates.

Additionally, the differences in diagnostic methods; the detection of different leishmania species; the sample sizes; the inclusion criteria; and the variation in the age, sex, and health statuses of the blood donors across the studies may have contributed to the observed heterogeneity. Differences in the sensitivity, specificity, and accuracy of diagnostic techniques (molecular and serological) can lead to discrepancies in parasite detection among blood donors. For example, the diagnosis of leishmaniasis in affected countries worldwide often relies on antibody-based tests, such as the rK39 strip test. However, these tests have limitations as they can read as positive in healthy individuals for extended periods, even after successful treatment. The molecular methods offer higher sensitivity and enable the early detection of parasites. In contrast, their use is often limited to skilled personnel and can be costly, making them less accessible in resource-limited and developing countries [6]. Therefore, it is essential to consider these factors when interpreting the prevalence estimates and addressing the heterogeneity observed. It is worth noting that significant heterogeneity is expected in the prevalence and incidence estimates due to this type of study. Consequently, it is important to note that a high I^2^ value in the context of proportional meta-analysis does not invariably signify data inconsistency [22].

To effectively implement these diagnostic tests in blood donation centers, several strategies are recommended. Establishing routine screening protocols that include both the serological and molecular tests can ensure comprehensive detection. Initial screening can be performed using serological methods, followed by confirmatory molecular tests for positive cases [4,6,27,33]. Training medical personnel in the use of these diagnostic methods and the interpretation of results is essential, along with continuous education programs to maintain high testing standards. Furthermore, ensuring that blood donation centers are equipped with the necessary infrastructure, such as PCR machines and trained laboratory technicians, is crucial for performing molecular diagnostics. Public health authorities can leverage the data from these diagnostic tests to develop guidelines and policies aimed at preventing the transmission of leishmaniasis through blood transfusions. This could include mandatory screening of all blood donors in endemic areas and the establishment of centralized testing facilities to standardize and streamline the screening process. By combining molecular and serological diagnostic methods, the risk of transmitting leishmaniasis through blood transfusions can be effectively mitigated, enhancing transfusion safety and public health [3,4,6,31,32].

To the best of our knowledge there are two meta-analyses regarding this issue to date. Asfaram S., et al. [57] conducted meta-analysis based on data from 16 studies involving 13,743 blood donors. They reported a prevalence rate of Leishmania infection of 7% (95% CI 5–8%) based on seropositivity, and a pooled prevalence rate of 2% (95%CI 1–3%) based on molecular tests. They also noted considerable heterogeneity between the studies. Another meta-analysis conducted by Foroutan M., et al. [58] analyzed data from 17,816 blood donors. They calculated the weighted overall prevalence of Leishmania infection as 4% (95% CI 2–7%) using serological methods and 8.7% (95% CI 4.2–14.3%) using molecular methods. Similar to the previous study, considerable heterogeneity was observed among the included studies.

Comparing the results of these meta-analyses with our own findings, we observed some variations in the estimated prevalence rates. These differences could be attributed to several factors, including variations in the number of included studies, the study populations, the geographical locations, the different inclusion/exclusion criteria, the quality assessment performed, the outlier and influential analyses performed, inherent heterogeneity among the studies themselves, and the potential temporal changes in the prevalence of leishmaniasis. Furthermore, it is important to consider the limitations of each study, including potential biases in sample selection and variations in the sensitivity and specificity of the diagnostic methods employed across studies. These factors may contribute to the observed variations in the prevalence estimates. Also, it is important to note that when considering the meta-analysis for of molecular method of diagnosis, we included studies that have implemented molecular diagnostic methods in the entire study population, rather than solely focusing on the individuals identified as positive through serological methods. This approach enables a more homogeneous population since a negative result from a serological method does not necessarily indicate a negative result from the molecular method.

Despite these variations, it is evident that leishmaniasis remains a significant public health concern among blood donors. In relation to the treatment of cutaneous leishmaniasis, it can be effectively addressed through various therapeutic approaches. The local therapy options encompass the utilization of pentavalent antimonials and paromomycin. Oral systemic therapy can be administered using azoles and miltefosine. For parenteral systemic therapy, it is recommended to employ pentavalent antimonials, amphotericin, and pentamidine [7,59]. Regarding the treatment of visceral leishmaniasis, it is advised to employ amphotericin B or pentavalent antimonian compounds [7]. Future research should focus on addressing the identified limitations and further investigating the factors influencing the prevalence of leishmaniasis to inform targeted interventions and control strategies [14,60,61].

### This Study’s Strengths and Limitations

The primary strength of this study lies in its thorough methodology, which included an extensive literature search, precise study selection, well-defined inclusion and exclusion criteria, eligibility screening, quality assessment, and the pooling analysis of prevalence data from thirty-five studies. However, it is important to note that significant unidentified heterogeneity persisted, requiring the careful interpretation of the results. The considerable variation in outcomes among the included studies was anticipated, given the nature of this type of research. Various factors, such as the differences in diagnostic methods, the detection of different leishmania species, the sample sizes, the inclusion criteria and the variation in the age, sex, the geographical distribution of cases, and the income levels and health status of blood donors across studies, may introduce bias when estimating the prevalence of leishmaniasis among blood donors. Furthermore, it should be noted that a positive diagnostic result does not necessarily indicate an active infection or the presence of an infected person and differences in the sensitivity, specificity, and accuracy of the diagnostic techniques (molecular and serological) can lead to discrepancies in parasite detection among blood donors. Due to insufficient data (fewer than ten studies for covariates) on variables like the proportion of males, mean age, and comorbidities, these factors were excluded from this analysis. Moreover, our meta-analysis has not been registered in PROSPERO, which may be a source of reporting bias. Additionally, only English language observational studies were included, leading to potential reporting bias. Our analysis ultimately included studies from Europe, South America, Asia, Africa, and Oceania. Consequently, it is crucial to interpret the results with caution, considering the limited generalizability of the data and the potential for underestimation or overestimation of prevalence.

## 5. Conclusions

In conclusion, our systematic review and meta-analysis on the prevalence of leishmaniasis among blood donors have shed light on this important public health concern. Our findings indicate a substantial prevalence of leishmaniasis among blood donors, as evidenced using both molecular and serological diagnostic methods, especially in endemic areas with a high incidence of parasitic diseases.

## Figures and Tables

**Table 1:** Descriptive characteristics of the included studies.

First Author	Year of Publication	Study Design	Continent of Origin	Country	Study Period	Total Blood Units	Proportion of Males (%)	Mean Age (years)	Leishmaniasis	Diagnostic Method	Quality Assessment
Mohammed R. [23]	2023	Cross-sectional	Africa	Ethiopia	2020	420	NA	NA	11	Molecular	High
						426	59	NA	31	Serological	High
Lopes E.A.D.O. [24]	2023	Cross-sectional	South America	Brazil	2018	324	73.5	NA	7	Serological	Moderate
Panahi E. [25]	2023	Cross-sectional	Oceania	Australia	2016	282	52.8	NA	96	Serological	Moderate
Naeem M.A. [26]	2023	Cross-sectional	Asia	Pakistan	2020	2000	100	NA	0	Molecular	High
Melkie I. [27]	2023	Cross-sectional	Africa	Ethiopia	2020	205	62.4	NA	32	Serological	Moderate
Rocha R. [28]	2023	Cross-sectional	Europe	Portugal	2022	3763		NA	201	Serological	High
Ortuno M. [29]	2022	Cross-sectional	Europe	Spain	2017–2018	550	53.5	NA	13	Molecular	Moderate
						670	53.5	NA	9	Serological	Moderate
Asfaram S. [30]	2022	Cross-sectional	Asia	Iran	2017–2018	860	98.5	35.1	388	Molecular	Moderate
						860	98.5	35.1	24	Serological	Moderate
Bayram G [31]	2020	Cross-sectional	Asia	Turkey	2016–2017	509	95	34	0	Serological	Moderate
						509	95	34	0	Molecular	Moderate
Ortalli M. [32]	2020	Cross-sectional	Europe	Italy	2014–2015	240	72.5	NA	4	Molecular	Moderate
						240	72.5	NA	27	Serological	Moderate
Silva L.P. [33]	2020	Cross-sectional	South America	Brazil	2017	500	57.4	NA	31	Molecular	Moderate
Abdon F.M. [34]	2020	Cohort	Africa	Sudan	NA	50	NA	NA	0	Molecular	Moderate
Aliaga L. [35]	2019	Cross-sectional	Europe	Spain	2015–2016	1189	NA	NA	30	Molecular	High
						1260	48.1	41	100	Serological	High
Ali S.O. [36]	2019	Cross-sectional	Africa	Sudan	2018	100	100	NA	0	Serological	Moderate
Ferroglio E. [37]	2018	Cross-sectional	Europe	Italy	NA	815	NA	NA	137	Serological	Moderate
Ferreira-Silva M.M. [38]	2018	Cross-sectional	South America	Brazil	2013–2014	615	NA	NA	44	Serological	Moderate
Asfaram S. [39]	2017	Cross-sectional	Asia	Iran	2016	600	99.3	39.3	23	Serological	Moderate
Timilsima S. [40]	2016	Cross-sectional	Asia	Nepal	2010	507	78.5	NA	5	Serological	Moderate
Banu S.S. [41]	2016	Cross-sectional	Asia	Bangladesh	2013–2014	706	NA	NA	9	Serological	Moderate
Monteiro D.C.S. [42]	2016	Cross-sectional	South America	Brazil	2011	431	NA	NA	57	Serological	Moderate
						431	NA	NA	20	Molecular	Moderate
Perez-Cutillas P. [43]	2015	Cross-sectional	Europe	Spain	2008–2010	618	46.6	NA	49	Molecular	Moderate
Sarkari B. [44]	2015	Cross-sectional	Asia	Iran	NA	2003	94.7	36.3	28	Serological	High
Braga L.D.S. [45]	2015	Cross-sectional	South America	Brazil	2011	176	65.3	NA	20	Serological	Moderate
Fukutani K.F. [46]	2014	Cross-sectional	South America	Brazil	2010	700	74.5	34	38	Serological	Moderate
Franca A.S.O. [47]	2013	Cross-sectional	South America	Brazil	2011	430	70.2	NA	67	Serological	Moderate
Huda M.M. [48]	2013	Cross-sectional	Asia	Bangladesh	2010–2011	1195	82	27.7	3	Serological	Moderate
Ates S.C. [49]	2013	Cross-sectional	Asia	Turkey	2008–2010	343	89	NA	12	Serological	Moderate
Chitimia L. [50]	2011	Cross-sectional	Europe	Spain	2008–2010	657	NA	NA	13	Serological	Moderate
						392	NA	NA	8	Molecular	Moderate
Riera C. [51]	2008	Cross-sectional	Europe	Spain	NA	304	NA	NA	18	Molecular	Moderate
						1437	NA	NA	44	Serological	Moderate
Scarlata F. [52]	2008	Cross-sectional	Europe	Italy	2005	1449	73	41	11	Serological	Moderate
Colomba C. [53]	2005	Cross-sectional	Europe	Italy	2002	500	NA	NA	0	Serological	Moderate
Kyriakou D.S. [54]	2003	Cross-sectional	Europe	Greece	NA	2000	41.6	NA	34	Molecular	Moderate
						2000	41.6	NA	304	Serological	Moderate
Fichoux Y.L. [55]	1999	Cross-sectional	Europe	Monaco	1996–1997	565	NA	NA	76	Serological	Moderate
Federico G. [56]	1991	Cross-sectional	Europe	Italy	NA	591	NA	NA	19	Serological	Moderate

NA: not applicable.

## Data Availability

Literature and Rstudio data are available from the corresponding author on reasonable request.

## Supplementary Materials

The following supporting information can be downloaded at: https://www.mdpi.com/article/10.3390/diseases12070160/s1, Table S1: PRISMA checklist; Table S2: Literature search; Figure S1: Visual representation of the influence diagnostics for each of the included studies regarding the prevalence of leishmaniasis among blood donors (molecular diagnosis); Figure S2: Forest plot displaying the re-calculated pooled effects, with one study omitted each time, using the leave-one-out method (molecular diagnosis); Figure S3: Visual representation of the influence diagnostics for each of the included studies regarding the prevalence of leishmaniasis among blood donors (serological diagnosis); Figure S4: Forest plot displaying the re-calculated pooled effects, with one study omitted each time, using the leave-one-out method (serological diagnosis).

## References

[1] Prevention CC for DC and CDC-Leishmaniasis-Epidemiology & Risk Factors. Published 27 February 2019. https://www.cdc.gov/leishmaniasis/risk-factors/index.html#:~:text=Places%20with%20increased%20risk,the%20outskirts%20of%20some%20cities.

[2] Georgiadou S.P., Makaritsis K.P., Dalekos G.N. (2015). Leishmaniasis revisited: Current aspects on epidemiology, diagnosis and treatment. J. Transl. Intern. Med..

[3] Maxfield L., Crane J.S. Leishmaniasis. PubMed. Published 2020. https://www.ncbi.nlm.nih.gov/books/NBK531456/.

[4] Mannan S.B., Elhadad H., Loc T.T., Sadik M., Mohamed M.Y., Nam N.H., Thuong N.D., Hoang-Trong B.L., Duc N.T., Hoang A.N. (2021). Prevalence and associated factors of asymptomatic leishmaniasis: A systematic review and meta-analysis. Parasitol. Int..

[5] Kantzanou M., Karalexi M.A., Theodoridou K., Kostares E., Kostare G., Loka T., Vrioni G., Tsakris A. (2023). Prevalence of visceral leishmaniasis among people with HIV: A systematic review and meta-analysis. Eur. J. Clin. Microbiol. Infect. Dis. Off. Publ. Eur. Soc. Clin. Microbiol..

[6] Thakur S., Joshi J., Kaur S. (2020). Leishmaniasis diagnosis: An update on the use of parasitological, immunological and molecular methods. J. Parasit. Dis..

[7] Prevention CC for DC and CDC-Leishmaniasis-Resources for Health Professionals. Published 13 June 2023.

[8] Aronson N., Herwaldt B.L., Libman M., Pearson R., Lopez-Velez R., Weina P., Carvalho E., Ephros M., Jeronimo S., Magill A. (2016). Diagnosis and Treatment of Leishmaniasis: Clinical Practice Guidelines by the Infectious Diseases Society of America (IDSA) and the American Society of Tropical Medicine and Hygiene (ASTMH). Am. J. Trop. Med. Hyg..

[9] Gow I., Smith N.C., Stark D., Ellis J. (2022). Laboratory diagnostics for human *Leishmania* infections: A polymerase chain reaction-focussed review of detection and identification methods. Parasites Vectors.

[10] Page M.J., McKenzie J.E., Bossuyt P.M., Boutron I., Hoffmann T.C., Mulrow C.D., Shamseer L., Tetzlaff J.T., Akl E.A., Brennan S.E. (2021). The PRISMA 2020 statement: An updated guideline for reporting systematic reviews. Br. Med. J..

[11] Zotero|Your Personal Research Assistant. https://www.zotero.org.

[12] França A.d.O., Pompilio M.A., Pontes E.R.J.C., Oliveira M.P., Pereira L.O.R., Lima R.B., Goto H., Sanchez M.C.A., Fujimori M., Lima-Júnior M.S.C. (2018). Leishmania infection in blood donors: A new challenge in leishmaniasis transmission?. PLoS ONE.

[13] A Newcastle-Ottawa Quality Assessment Scale (Adapted for Cross Sectional Studies). https://cdn-links.lww.com/permalink/ejgh/a/ejgh_31_9_2019_07_18_nguyen_15743_sdc1.pdf.

[14] Kantzanou M., Kostares E., Kostare G., Boufidou F., Tzanai A., Kostares M., Tsakris A. (2024). Prevalence of ocular toxoplasmosis among people living with HIV: A systematic review and meta-analysis. Futur. Microbiol..

[15] RStudio Desktop. Posit. https://posit.co/download/rstudio-desktop.

[16] Viechtbauer W. (2010). Conducting Meta-Analyses in R with the metafor package. J. Stat. Softw..

[17] Miller J.J. (1978). The Inverse of the Freeman–Tukey Double Arcsine Transformation. Am. Stat..

[18] Viechtbauer W., Cheung M.W.L. (2010). Outlier and influence diagnostics for meta-analysis. Res. Synth. Methods.

[19] Ensure That There Are Adequate Studies. https://handbook-5-1.cochrane.org/chapter_9/9_6_5_1_ensure_that_there_are_adequate_studies_to_justify.htm.

[20] Egger M., Smith G.D., Schneider M., Minder C. (1997). Bias in meta-analysis detected by a simple, graphical test. BMJ.

[21] Begg C.B., Mazumdar M. (1994). Operating characteristics of a rank correlation test for publication bias. Biometrics.

[22] Barker T.H., Migliavaca C.B., Stein C., Colpani V., Falavigna M., Aromataris E., Munn Z. (2021). Conducting proportional meta-analysis in different types of systematic reviews: A guide for synthesisers of evidence. BMC Med. Res. Methodol..

[23] Mohammed R., Melkamu R., Pareyn M., Abdellati S., Bogale T., Engidaw A., Kinfu A., Girma T., Van Griensven J. (2023). Detection of asymptomatic Leishmania infection in blood donors at two blood banks in Ethiopia. PLoS Negl. Trop. Dis..

[24] Lopes E.A.D.O., Florencio-Henschel P., Jordão F.T., Sperança M.A., Martins L.P.A., Suzuki R.B. (2023). Leishmania infantum (syn. Leishmania chagasi) detection in blood donors living in an endemic area. Parasitol. Res..

[25] Panahi E., Stanisic D.I., Skinner E.B., Faddy H.M., Young M.K., Herrero L.J. (2023). Detection of Leishmania (Mundinia) macropodum (Kinetoplastida: Trypanosomatidae) and heterologous Leishmania species antibodies among blood donors in a region of Australia with marsupial Leishmania endemicity. Int. J. Infect. Dis..

[26] Abdul Naeem M., Aamir M., Ijaz F., Amin N., Khurram Aftab R. (2023). Detection of asymptomatic Leishmania donovani in healthy voluntary blood donors. Transfus. Clin. Et. Biol..

[27] Melkie I., Yimer M., Alemu G., Tegegne B. (2023). Asymptomatic Leishmania donovani infection and associated factors among blood donors attending at Metema district Blood Bank, Northwest Ethiopia: A cross- sectional study. Arch. Public Health.

[28] Rocha R., Gonçalves L., Conceição C., Andrade P., Cristóvão J.M., Condeço J., Delgado B., Caeiro C., Kuzmenko T., Vasconcelos E. (2023). Prevalence of asymptomatic Leishmania infection and knowledge, perceptions, and practices in blood donors in mainland Portugal. Parasites Vectors.

[29] Ortuño M., Muñoz C., Spitzová T., Sumova P., Iborra M.A., Pérez-Cutillas P., Ayhan N., Charrel R.N., Volf P., Berriatua E. (2022). Exposure to Phlebotomus perniciosus sandfly vectors is positively associated with Toscana virus and Leishmania infantum infection in human blood donors in Murcia Region, southeast Spain. Transbounding Emerg. Dis..

[30] Asfaram S., Fakhar M., Mohebali M., Ziaei Hezarjaribi H., Mardani A., Ghezelbash B., Akhoundi B., Zarei Z., Moazeni M. (2022). A Convenient and Sensitive kDNA-PCR for Screening of Leishmania infantum Latent Infection Among Blood Donors in a Highly Endemic Focus, Northwestern Iran. Acta Parasit..

[31] Bayram G., Dinçer E., Erden Ertürk S., Tiftik E.N. (2020). Seroprevalence of Asymptomatic Leishmania spp. Carriage Among Blood Donors in Leishmaniasis Endemic Area in Turkey. Flora J. Infect. Dis. Clin. Microbiol..

[32] Ortalli M., De Pascali A.M., Longo S., Pascarelli N., Porcellini A., Ruggeri D., Randi V., Procopio A., Re M.C., Varani S. (2020). Asymptomatic Leishmania infantum infection in blood donors living in an endemic area, northeastern Italy. J. Infect..

[33] Silva L.P., Montenegro S., Werkauser R., Sales K.G.D.S., Soares F.C.S., Costa V.M.A., Bezerra A.C., Pinto M.B.D.A., Ferreira S.M., Neitzke-Abreu H.C. (2020). Asymptomatic Leishmania infection in blood donors from a major blood bank in Northeastern Brazil: A cross-sectional study. Rev. Inst. Med. Trop. Sao Paulo.

[34] Abdon F.M., Abbadi O.S., Saad A.A. (2020). Testing the prevalence of Leishmania Donovani DNA in the Blood of Sudanese blood donors. Infect. Dis. Trop. Med..

[35] Aliaga L., Ceballos J., Sampedro A., Cobo F., López-Nevot M.Á., Merino-Espinosa G., Morillas-Márquez F., Martín-Sánchez J. (2019). Asymptomatic Leishmania infection in blood donors from the Southern of Spain. Infection. Infection.

[36] Ali S.O. (2019). The Incidence of Leishmania donovani using RK39 and Buffy Coat Concentration Technique in Blood Donors in Gadarif State. Int. J. Med. Res. Health Sci..

[37] Ferroglio E., Battisti E., Zanet S., Bolla C., Concialdi E., Trisciuoglio A., Khalili S., Biglino A. (2018). Epidemiological evaluation of Leishmania infantum zoonotic transmission risk in the recently established endemic area of Northwestern Italy. Zoonoses Public Health.

[38] Ferreira-Silva M.M., Teixeira L.A.S., Tibúrcio M.S., Pereira G.A., Rodrigues V., Palis M., Afonso P., Alves M., Feitosa J.M., Urias E. (2018). Socio-epidemiological characterisation of blood donors with asymptomatic *Leishmania infantum* infection from three Brazilian endemic regions and analysis of the transfusional transmission risk of visceral leishmaniasis. Transfus. Med..

[39] Asfaram S., Fakhar M., Mohebali M., Mardani A., Banimostafavi E.S., Ziaei Hezarjaribi H., Soosaraei M. (2017). Asymptomatic human blood donors carriers of Leishmania infantum: Potential reservoirs for visceral leishmaniasis in northwestern Iran. Transfus. Apher. Sci..

[40] Timilsina S., Raj Bhattarai N., Khanal B., Rijal S. (2016). Serological Assessment for Leishmania donovani Infection in Blood Donors of Sunsari District, Dharan, Nepal. Indian J. Hematol. Blood Transfus..

[41] Banu S.S., Ahmed B.-N., Shamsuzzaman A.K.M., Lee R. (2016). Evaluation of recombinant K39 antigen and various promastigote antigens in sero-diagnosis of visceral leishmaniasis in Bangladesh. Parasite Epidemiol. Control..

[42] Monteiro D.C., Sousa A.Q., Lima D.M., Fontes R.M., Praciano C.C., Frutuoso M.S., Matos L.C., Teixeira M.J., Pearson R.D., Pompeu M.M. (2016). *Leishmania infantumInfection* in Blood Donors, Northeastern Brazil. Emerg. Infect. Dis..

[43] Pérez-Cutillas P., Goyena E., Chitimia L., De La Rúa P., Bernal L.J., Fisa R., Riera C., Iborra A., Murcia L., Segovia M. (2015). Spatial distribution of human asymptomatic Leishmania infantum infection in southeast Spain: A study of environmental, demographic and social risk factors. Acta Trop..

[44] Sarkari B., Gadami F., Shafiei R., Motazedian M.H., Sedaghat F., Kasraian L., Tavasoli A.R., Zarnegar G., Nikmanesh Y., Davami M.H. (2015). Seroprevalence of Leishmania infection among the healthy blood donors in kala-azar endemic areas of Iran. J. Parasit. Dis..

[45] Braga L.D.S., Navasconi T.R., Leatte E.P., Skraba C.M., Silveira T.G.V., Ribas-Silva R.C. (2015). Presence of anti-Leishmania (Viannia) braziliensis antibodies in blood donors in the West-Central region of the State of Paraná, Brazil. Rev. Soc. Bras. Med. Trop..

[46] Fukutani K.F., Figueiredo V., Celes F.S., Cristal J.R., Barral A., Barral-Netto M., De Oliveira C.I. (2014). Serological survey of Leishmaniainfection in blood donors in Salvador, Northeastern Brazil. BMC Infect. Dis..

[47] França A.d.O., Castro V.L.d., Junior M.S.d.C.L., Pontes E.R.J.C., Dorval M.E.C. (2013). Anti-Leishmania antibodies in blood donors from the Midwest region of Brazil. Transfus. Apher. Sci..

[48] Huda M.M., Rudra S., Ghosh D., Bhaskar K.R.H., Chowdhury R., Dash A.P., Bhattacharya S.K., Haque R., Mondal D. (2013). Low prevalence of Leishmania donovani infection among the blood donors in kala-azar endemic areas of Bangladesh. BMC Infect. Dis..

[49] Ates S.C., Bagirova M., Allahverdiyev A.M., Baydar S.Y., Koc R.C., Elcicek S., Abamor E.S., Oztel O.N. (2012). Detection of antileishmanial antibodies in blood sampled from blood bank donors in Istanbul. Future Microbiol..

[50] Chitimia L., Muñoz-García C.I., Sánchez-Velasco D., Lizana V., Del Río L., Murcia L., Fisa R., Riera C., Giménez-Font P., Jiménez-Montalbán P. (2011). Cryptic Leishmaniosis by Leishmania infantum, a feature of canines only? A study of natural infection in wild rabbits, humans and dogs in southeastern Spain. Vet. Parasitol..

[51] Riera C., Fisa R., Lpez-Chejade P., Serra T., Girona E., Jimnez M., Muncunill J., Sedeo M., Mascar M., Udina M. (2008). Asymptomatic infection by Leishmania infantum in blood donors from the Balearic Islands (Spain). Transfusion.

[52] Scarlata F., Vitale F., Saporito L., Reale S., Vecchi V.L., Giordano S., Infurnari L., Occhipinti F., Titone L. (2008). Asymptomatic Leishmania infantum/chagasi infection in blood donors of western Sicily. Trans. R. Soc. Trop. Med. Hyg..

[53] Colomba C., Saporito L., Polara V.F., Barone T., Corrao A., Titone L. (2005). Serological screening for Leishmania infantum in asymptomatic blood donors living in an endemic area (Sicily, Italy). Transfus. Apher. Sci..

[54] Kyriakou D.S., Alexandrakis M.G., Passam F.H., Kourelis T.V., Foundouli P., Matalliotakis E., Maniatis A.N. (2003). Quick detection of Leishmania in peripheral blood by flow cytometry. Is prestorage leucodepletion necessary for leishmaniasis prevention in endemic areas?. Transfus. Med..

[55] Le Fichoux Y., Quaranta J.F., Aufeuvre J.P., Lelievre A., Marty P., Suffia I., Rousseau D., Kubar J. (1999). Occurrence of Leishmania infantum Parasitemia in Asymptomatic Blood Donors Living in an Area of Endemicity in Southern France. J. Clin. Microbiol..

[56] Federico G., Damiano F., Caldarola G., Fantini C., Fiocchi V., Ortona L. (1991). A seroepidemiological survey on Leishmania infantum infection. Eur. J. Epidemiol..

[57] Asfaram S., Fakhar M., Soosaraei M., Teshnizi S.H., Mardani A., Banimostafavi E.S., Hezarjaribi H.Z. (2017). Global status of visceral leishmanial infection among blood donors: A systematic review and meta-analysis. Transfus. Apher. Sci..

[58] Foroutan M., Dalvand S., Khademvatan S., Majidiani H., Khalkhali H., Masoumifard S., Shamsaddin G. (2017). A systematic review and meta-analysis of the prevalence of Leishmania infection in blood donors. Transfus. Apher. Sci..

[59] Goto H., Lauletta Lindoso J.A. (2012). Cutaneous and Mucocutaneous Leishmaniasis. Infect. Dis. Clin. N. Am..

[60] Schistosomiasis. https://www.who.int/news-room/fact-sheets/detail/schistosomiasis#:~:text=Schistosomiasis%20is%20an%20acute%20and%20chronic%20parasitic%20disease%20caused%20by.

[61] Kantzanou M., Karalexi M.A., Vassalos C.M., Kostare G., Vrioni G., Tsakris A. (2022). Central nervous system cystic echinococcosis: A systematic review. Germs.

